# Formulation and Characterization of Nanoemulsion Incorporating *Chamomilla recutita* L. Extract Stabilized with Hyaluronic Acid

**DOI:** 10.3390/pharmaceutics16060701

**Published:** 2024-05-23

**Authors:** Getulio Capello Tominc, Mariana Dalmagro, Elton da Cruz Alves Pereira, Maisa Steffani Adamczuk, Francieli Gesleine Capote Bonato, Rafael Menck de Almeida, Ricardo Schneider, Melyssa Fernanda Norman Negri, Daniela Dib Gonçalves, Jaqueline Hoscheid

**Affiliations:** 1 Graduate Program in Medicinal Plants and Herbal Medicines in Basic Health Care, Universidade Paranaense (UNIPAR), Umuarama 87502-210, Brazil; getulio.tominc@edu.unipar.br; 2 Graduate Program in Biotechnology Applied to Agriculture, Universidade Paranaense (UNIPAR), Umuarama 87502-210, Brazil; mariana.dal@edu.unipar.br (M.D.); elton.221872@edu.unipar.br (E.d.C.A.P.); 3 Graduate Program in Health Sciences, State University of Maringa, Maringa 87080-000, Brazil; mfnngrassi2@uem.br; 4 Graduate in Pharmacy, Universidade Paranaense (UNIPAR), Toledo 85903-170, Brazil; maisa.adamczuk@edu.unipar.br; 5 Graduate Program in Animal Science with Emphasis on Bioactive Products, Universidade Paranaense (UNIPAR), Umuarama 87502-210, Brazil; francieli.bonato@edu.unipar.br (F.G.C.B.); danieladib@prof.unipar.br (D.D.G.); 6 Synthetica Research and Technical Analysis Ltda., Capela do Alto, São Paulo 18195-000, Brazil; rafaelmenck@synthetica.com.br; 7 Group of Polymers and Nanostructures, Federal Technological University of Paraná, Toledo 85902-490, Brazil; rschneider@utfpr.edu.br

**Keywords:** antibacterial activity, chamomile, emulsion, skin infection, skin lesion

## Abstract

Skin lesions are an important health concern, exposing the body to infection risks. Utilizing natural products containing chamomile (*Chamomilla recutita* L.) holds promise for curative purposes. Additionally, hyaluronic acid (HA), an active ingredient known for its tissue regeneration capacity, can expedite healing. In this study, we prepared and characterized an extract of *C. recutita* and integrated it into a nanoemulsion system stabilized with HA, aiming at harnessing its healing potential. We assessed the impact of alcoholic strength on flavonoid extraction and chemically characterized the extract using UHPLC/MS while quantifying its antioxidant and antimicrobial capacity. We developed a nanoemulsion loaded with *C. recutita* extract and evaluated the effect of HA stabilization on pH, droplet size, polydispersity index (PDI), zeta potential, and viscosity. Results indicated that 70% hydroalcoholic extraction yielded a higher flavonoid content. The extract exhibited antioxidant capacity in vitro, a desirable trait for skin regeneration, and demonstrated efficacy against key microbial strains (*Staphylococcus aureus*, *Streptococcus pyogenes*, *Escherichia coli*, and *Pseudomonas aeruginosa*) associated with skin colonization and infections. Flavonoids spireoside and apiin emerged as the most abundant bioactives. The addition of HA led to increased viscosity while maintaining a suitable pH for topical application. Zeta potential, droplet size, and PDI met acceptable criteria. Moreover, incorporating *C. recutita* extract into the nanoemulsion enhanced its antimicrobial effect. Hence, the nanoemulsion system loaded with *C. recutita* and HA stabilization exhibits favorable characteristics for topical application, showing promise in aiding the healing processes.

## 1. Introduction

Skin trauma, characterized by interruptions in skin-mucosal integrity, serves as a potential entry point for bacterial infections, significantly impacting individuals’ lives and causing socioeconomic disruptions [1]. A growing body of evidence suggests that natural products hold promise in treating some skin diseases. This is due to their diverse pharmacological effects, notably anti-inflammatory, antioxidant, and antimicrobial properties, alongside good tolerability and safety, which contribute synergistically to tissue repair [2].

For thousands of years, the floral heads of chamomile (*Chamomilla recutita* L., *Matricaria recutita* L., or *Matricaria chamomilla*) have been employed in traditional and folk medicine via infusion, attributed to their antioxidant, anti-inflammatory, and antibacterial properties, particularly associated with the flavonoids present in the extract. These properties have facilitated its use across pharmaceutical, cosmetic, and food industries [3]. Moreover, chamomile oil extract has demonstrated efficacy in accelerating tissue repair post-skin injury [4] and burns [5] in in vivo models.

Additionally, the distinctive physicochemical properties and multifaceted physiological functions of hyaluronic acid (HA), a primary component of the skin’s extracellular matrix, have rendered it invaluable in regenerative medicine and tissue engineering, showing promising applications in skin tissue repair [6]. Recent advancements in the treatment and prevention of bacterial infections, leveraging nanomaterials, have underscored the efficacy of combining HA with antibacterial agents to overcome the limitations of traditional antibiotics in infection management and enhance the resolution of chronic wounds [7]. Hence, this study undertook the preparation, characterization, and integration of *C. recutita* extract into a nanoemulsion system stabilized with HA, aiming to augment tissue repair processes.

## 2. Materials and Methods

### 2.1. Chemicals

Sodium dodecyl sulfate, sorbitan monooleate (Span 80), polyoxyl 40 hydrogenated castor oil (Cremophor^®^ RH40), aluminum chloride, 2,2-diphenyl-1-picryhydrazyl (DPPH), 2,2-azinobis(3-ethylbenzthiazoline-6-sulfonic acid) (ABTS), 2,4,6-Tris(2-pyridyl)-s-triazine (TPZT), Trolox, triphenyl tetrazolium chloride (TTC), and Folin-Ciocalteu reagent were obtained from Sigma-Aldrich^®^ (St. Louis, MO, USA). Hyaluronic acid (HA) (molecular weight: 1.21 × 10^6^ g/mol; purity: 95.7%) was purchased from Hyaxel^®^ (São Paulo, SP, Brazil). Corn oil and sodium carbonate (Na_2_CO_3_) were acquired from Liza (Mairinque, SP, Brazil) and Êxodo Científica (Sumaré, SP, Brazil), respectively. Brain heart infusion (BHI) broth and Neomycin were obtained from Acumedia^®^ (Lansing, MI, USA) and Sovitá Ativos Company (São Paulo, SP, Brazil), respectively. Methyl alcohol and ethyl alcohol P.A. were purchased from Synth^®^ (Diadema, SP, Brazil). All other reagents were of analytical grade.

### 2.2. Preparation of Chamomilla Recutita Extract

Floral heads of *C. recutita* sourced from Mandirituba (Paraná State, Brazil), harvested in August 2021, were procured from a supplier of botanical raw materials in Cascavel (Paraná State, Brazil), along with a quality assurance report.

The material was pulverized in a knife mill (TE 631/2, TECNAL, Piracicaba, São Paulo State, Brazil) to achieve a particle size of 0.180 mm and used for extractions at various alcoholic concentrations (50, 60, 70, 80, 90, and 100%), employing a plant/solvent ratio of 1:10 (*w*/*v*). Extraction was conducted by vortex extraction in a high-speed shear apparatus (Ultra-Turrax^®^ T-25, IKA, Wilmington, NC, USA) for 5 min at 9000 rpm and 25 °C. Subsequently, the extracts were filtered, and ethanol was removed using a rotary evaporator (RV 10, IKA, Wilmington, NC, USA) at 60 °C, 180 mbar pressure, and 180 rpm. The resultant extracts were then frozen and freeze-dried (model LJJ02, JJ Científica, São Paulo, SP, Brazil) until dry.

Concurrently, conventional extraction was performed via infusion, maintaining a plant/solvent ratio of 1:10. Boiling water was added, and the system was allowed to rest for 30 min, followed by filtration, freezing, and freeze-drying.

To determine the optimal extraction conditions, the total flavonoid content (TFT) of all extracts was quantified using a spectrophotometer (Model IL-592, Kasuaki, São Paulo, SP, Brazil) at 425 nm, following the methodology outlined by Woisky and Salatino [8]. Quercetin served as the standard, providing the equation for the calibration curve: y = 81.561x − 126.41 (R^2^ = 0.9966). Analyses were performed in triplicate, and the results were expressed as µg quercetin equivalents per gram of extract (µg_QUE_ mg_ext_^−1^).

### 2.3. Extract Characterization

#### 2.3.1. Phytochemical Identification by Ultra-High Pressure Liquid Chromatograph with a Mass Spectrometer (UHPLC-MS)

Chromatographic profiling and identification were conducted using an ultra-high-pressure liquid chromatograph (UHPLC) equipped with a BEH C-18 water absorption column (150 mm × 2.1 mm × 1.7 μm), coupled to a mass spectrometer (MS) featuring a quadrupole-time-of-flight system (BRUKER, Q-TOFII^®^, Billerica, MA, USA). Analyses were carried out in both positive and negative modes, following the conditions outlined by Dalmagro et al. [9].

For identification purposes, chromatograms were imported into MetaboScape software (https://www.bruker.com/en/products-and-solutions/mass-spectrometry/ms-software/metaboscape.html, accessed on 19 May 2024, Bruker^®^, Billerica, MA, USA) and compared with various databases (Bruker MetaboBASE^®^ Personal Library 3.0, Bruker HMDB Metabolite Library 2.0, and Bruker MetaboBASE^®^ Plant Library, MA, USA). Peaks with a minimum intensity of 1000 were observed with mSigma set at 20.

#### 2.3.2. Antioxidant Capacity

The extract, at a concentration of 1000 µg mL^−1^, underwent triplicate evaluation for its capacity to eliminate the radicals DPPH and ABTS^•+^, as well as its reducing capacity via the FRAP assay, using a spectrophotometer.

For the DPPH method, a calibration curve (50–1000 μM) was constructed employing Trolox as the standard (y = −0.5771x + 673.63; R^2^ = 0.9968), with the DPPH radical scavenging capacity expressed in μM Trolox equivalent (μM_Trolox_) [10]. The ABTS^•+^ free radical scavenging assay followed the protocol outlined by Re et al. [11]. A calibration curve (100–2000 μM) was generated using Trolox as the standard (y = −0.2465x + 750.59; R^2^ = 0.9914), and the ABTS^•+^ free radical scavenging capacity was reported in μmol of Trolox per gram of extract (µmol_Trolox_ gext^−1^). The FRAP assay estimated the reducing capacity based on a calibration curve (y = 0.6188x − 96.833; R^2^ = 0.9926) for ferrous sulfate (100–2000 µM), with results presented as µmol of Fe^2+^ per gram of extract (µmolFe^2+^ gext^−1^), following the methodology outlined by Santos et al. [12].

### 2.4. Nanoemulsion System Loaded with C. recutita and Stabilized with Hyaluronic Acid Development

The nanoemulsion preparation involved two steps. Firstly, a pre-emulsion was prepared by blending 0.1 M saline solution (22.0%, *w/w*), sodium dodecyl sulfate (2.5%, *w/w*), and chamomile extract (1.0%, *w/w*), followed by gradual addition of this mixture to a blend of corn oil (70%, *w/w*) and Span 80 (4.5%, *w/w*) through dripping, using a high-speed shearing apparatus at 9000 rpm for 300 s. The resulting pre-emulsion underwent sonication using an ultrasound device equipped with a 13 mm diameter ultrasonic tip (Eco-Sonics, Indaiatuba, SP, Brazil), operating at a frequency of 20 kHz and power of 80%, for 60 s [13]. Concentrations were selected based on pre-formulation studies encompassing the lower and upper limits of each variable.

In the second stage, the pre-emulsion was dripped into an aqueous phase consisting of a surfactant agent (Cremophor RH 40) and 0.1 M saline solution, as outlined in Table 1, using a high-speed shearing device at 9000 rpm for 300 s. Hyaluronic acid was subsequently introduced, and stirring was continued for 2 h at room temperature (25 °C) employing a magnetic stirrer [14]. The control formulation followed identical procedures but omitted the incorporation of *C. recutita* extract into the pre-emulsion. All preparations were conducted in triplicate and left to stand for 24 h at 25 ± 1 °C before characterization. The selection of surfactant was based on previous studies confirming its safety and non-toxicity [15,16].

### 2.5. Nanoemulsion Systems Characterization

#### 2.5.1. Droplet Size and Polydispersity Index

For droplet size and polydispersity index (PDI) measurements, 20 µL of samples were dispersed in 20 mL of 0.1 M NaCl solution at the time of analysis. The refractive index of the oil phase was 1.420. Analyses were conducted using a laser diffraction particle size analyzer (Partica LA-960, HORIBA Scientific, Piscataway, NJ, USA), with an evaluation range from 10 nm at 5 mm, at 25 ± 1 °C, in triplicate. Results were expressed as the mean ± standard deviation.

#### 2.5.2. pH

pH was measured in triplicate using a potentiometer calibrated at 25 ± 1 °C (Ionlab^®^, PHB 500, Araucária, PR, Brazil).

#### 2.5.3. Viscosity

Viscosity was measured at speeds ranging from 1 to 40 rpm using number 2 cylindrical spindles, with readings limited to the maximum speed, in a digital Brookfield viscometer (QUIMIS^®^, model Q860M26, Diadema, SP, Brazil). The instrument provided precise readings of ±2.0% with a measurement range of 1 to 6,000,000 mPa s, at 25 ± 1 °C [17].

#### 2.5.4. Zeta Potential

Surface charge was determined by electrostatic mobility using a particle analyzer (Malvern Panalytical, Malvern, UK) at 25 ± 1 °C. Aliquots were diluted 1:200 with NaCl (pH = 7.40 ± 0.05) immediately before analysis. Zeta potential was calculated using the Helmholtz–Smoluchowski equation [18].

### 2.6. Morphology

For morphological evaluation, the F2 + HA nanoemulsion was placed on a 300-mesh copper grid coated with carbon film and negatively stained with a 2% phosphotungstic acid solution. The grids were air-dried for 24 h at 25 ± 1 °C [19], and images were captured using transmission electron microscopy (TEM) (JEOL JEM 1400 TEM, Peabody, MA, USA).

### 2.7. In Vitro Antimicrobial Activity

The minimum inhibitory concentration (MIC) was determined using the serial microdilution method in 96-well plates, in triplicate, following a previously described methodology [9]. The test was performed against the following microorganisms: *Staphylococcus aureus* (ATCC 12026), *Streptococcus pyogenes* (ATCC 19615), *Escherichia coli* (ATCC 25922), and *Pseudomonas aeruginosa* (ATCC 9027). The positive control comprised the first three lines containing broth, microorganisms, and commercial antimicrobial (neomycin at concentrations ranging from 1.22 × 10^−3^ to 2.5 mg mL^−1^). The negative control consisted of the last line containing broth and microorganisms. The *C. recutita* extract, as well as the nanoemulsions control (C2 and C2 + HA) and those loaded with extract (F2 and F2 + HA), were added to microplates containing BHI at concentrations ranging from 0.24 to 500 mg mL^−1^. The plates were then incubated at 36 ºC for 24 h, after which 2% TTC developer was added and the appearance of a pink color was observed, indicating bacterial growth.

### 2.8. Statistical Analysis

The results were subjected to analysis of variance (ANOVA) and compared using the Tukey test at a significance level of 5%, using the STATISTICA 13.0 program (Statsoft^®^, Tulsa, OK, USA).

## 3. Results

This study aimed to prepare, characterize, and integrate *C. recutita* extract into a nanoemulsion system stabilized with HA, with the potential to combat bacterial infections and facilitate differential healing of skin lesions. Different alcoholic contents were employed to optimize the extraction of flavonoids (Table 2).

The 70% ethanol extraction yielded a higher TFT, and the extract obtained under this condition underwent phytochemical characterization by UHPLC-MS (Table 3). This extract, at a concentration of 1000 µg mL^−1^, exhibited scavenging capacities for DPPH and ABTS^•+^ radicals of 22.324 ± 0.36 μM_Trolox_ and 778.593 ± 27.01 µmol_Trolox_ g_ext_^−1^, respectively. Additionally, the reduction capacity of FRAP was measured at 1085.702 ± 23.31 µmol_Fe_^2+^ g_ext_^−1^.

Smaller droplet sizes and PDI values (Table 4) were attained with a 10.0% surfactant concentration (hydrophilic–lipophilic balance = 11.37). Incorporating the *C. recutita* extract did not cause a statistically significant difference in droplet size or PDI, compared to the corresponding control, indicating that the extract does not interfere with nanoemulsion formation. The systems exhibited pH levels compatible with topical application and satisfactory zeta potential.

**Figure 1:**
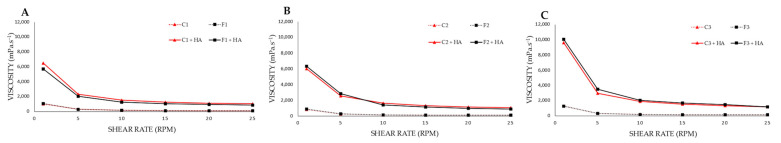
Surfactant concentration effect on the rheology of a nanoemulsion system loaded with *C. recutita* extract and stabilized with hyaluronic acid. (**A**) Nanoemulsions with 7.5% surfactant; (**B**) nanoemulsions with 10.0% surfactant; and (**C**) nanoemulsions with 12.5% surfactant. C: nanoemulsions system without *C. recutita* extract. F: nanoemulsions system loaded with *C. recutita* extract. HA: hyaluronic acid for stabilization.

The incorporation of HA notably elevated the viscosity of the nanoemulsion systems compared to those not stabilized with HA. Moreover, higher surfactant concentrations in HA-stabilized systems resulted in a slight increase in resting viscosity. Rheogram analysis revealed pseudoplastic flow behavior (Figure 1), confirming the heightened viscosity in nanoemulsions stabilized with HA. Importantly, the addition of the extract did not exert a significant influence on viscosity parameters.

**Figure 2:**
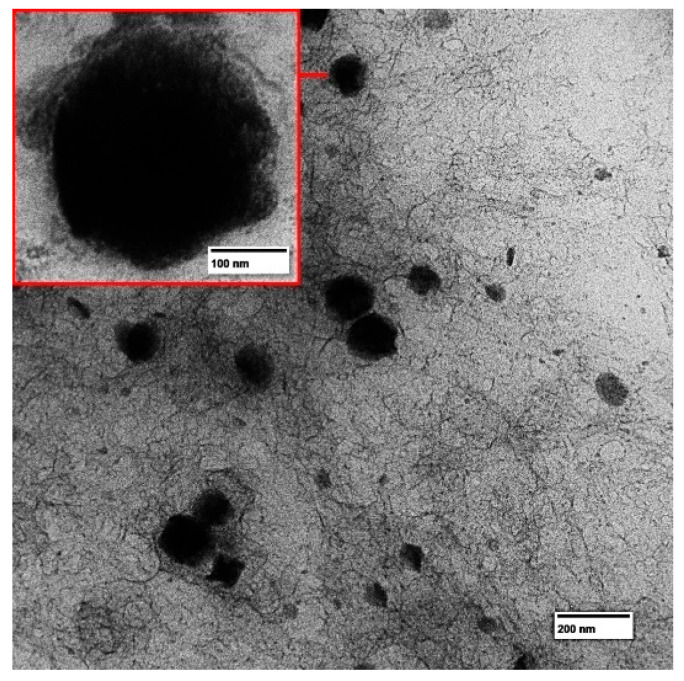
Photomicrographs of F2 + HA at 120 kV (magnification: 50 k).

To elucidate the morphology and validate the droplet size, the nanoemulsion exhibiting satisfactory characteristics (F2 + HA) was examined via TEM. As depicted in Figure 2, the nanoemulsion displays a spherical shape with a narrow distribution. Additionally, the presence of HA chains surrounding the droplets was observed. Importantly, the droplet size observed aligns with the data obtained from laser diffraction.

In conclusion, the assessment of MIC affirmed the efficacy of *C. recutita* extract and nanoemulsions loaded (F2) and stabilized with HA (F2 + HA) in inhibiting bacterial multiplication (Table 5). It is noteworthy that the MIC for F2 denotes the concentration (mg mL^−1^) of nanoemulsion, where 250 mg is equivalent to 0.5 mg of *C. recutita* extract, indicating the augmentation of the antibacterial effect of the extract when incorporated into the nanoemulsion system. Additionally, the stabilization of the system with HA reduced the MIC against *S. aureus* and *P. aeruginosa* (MIC equivalent to 0.333 mg of extract). As anticipated, nanoemulsions (C2 and C2 + HA) lacking *C. recutita* extract demonstrated no inhibitory activity.

## 4. Discussion

The vortical extraction method demonstrated a significantly higher potential for flavonoid extraction when using 70% ethanol. This observation is consistent with findings by Weber et al. [20], who illustrated superior extraction outcomes for chamomile using hyphenation techniques at the same ethanol concentration. Additionally, Asadi et al. [21] reported that *C. recutita* extract in 70% ethanol affects macrophages, promoting the reduction of nitric oxide synthesis and displaying anti-inflammatory properties. This activity was attributed to apigenin, which was also identified during the chemical characterization of our extract.

Furthermore, among the identified compounds, luteolin, naringin, kaempferide, and its conjugates, as well as salicylic acid, are commonly encountered [22,23,24,25,26]. Evidence suggests that spireoside, the predominant component, functions as an effective antioxidant and anti-inflammatory agent in wound healing by reducing reactive oxygen species (ROS) [27].

Flavonoids comprise the majority class in the floral heads of *C. recutita*. These compounds are directly linked to antioxidant, cytotoxic, anti-allergic, analgesic, and bactericidal activities, as well as acceleration of the healing process [28,29,30]. Studies on structure-activity relationships have identified several factors potentially responsible for the high antioxidant activity of flavonoids. These factors include the presence of hydroxyl groups at positions 3 and 5 of ring A, position and quantity of –OH in ring B, degree of methylation of 3–OH, the double bond between carbons 2–3 in conjugation, presence of the 4–oxo function in the C ring, and angulation of flavonoid skeleton [31,32].

Furthermore, after a skin injury, ROS over production during the inflammatory phase can induce damage and hinder wound healing [33]. Hence, the application of *C. recutita* extract offers a promising alternative as a natural product in the development of topical delivery systems. Moreover, the extract has demonstrated potential antimicrobial activity, fostering a conducive environment for the healing process.

Flavonoids are well known for their antimicrobial effects through various mechanisms. For example, apigenin disorients the lipid components of the membrane, leading to cell disruption [34]. Additionally, apigenin, naringenin, kaempferol, chrysin, and quercetin interfere with biofilm formation [35]. Moreover, epicatechin and quercetin, along with their glycosylated derivatives such as spireoside, induce oxidative damage to the membrane, increasing cellular permeability [36]. Furthermore, quercetin and luteolin inhibit bacterial DNA replication [34].

Notably, variations in surfactant concentration during the nanoemulsion system development cause changes in droplet size and PDI. As the concentration of surfactant agent increases up to 10.0%, particle size and PDI gradually decrease. This is because surfactants play a crucial role in maintaining the stability and resistance of nanoemulsion structures to variations [37]. However, adding surfactants at concentrations above the ideal leads to particle entanglement, thereby destabilizing the system [38]. This observation aligns with the data presented here, wherein a concentration of 12.5% surfactant increased in size and droplet PDI.

In general, most nanoemulsions without HA exhibited a narrower size distribution. The increase in droplet diameter and PDI in the presence of HA can be attributed to the presence of HA chains at the interface of the oil droplets and the formation of surfactant–HA aggregates on the interfacial surface of the droplet [14]. Despite the increase in PDI, the values remained below 0.3, which is considered a narrow distribution and indicates homogeneous droplets [19,39]. Although coalescence is an unfavorable phenomenon for emulsifier systems and occurs in formulations with high PDI, it is important to note that PDI is only one of the general factors that influence stability. Therefore, despite the increase in PDI, HA also increased viscosity, reducing Brownian movement and collision between droplets and consequently protecting the coalescence system [14].

The viscosity increase resulting from HA inclusion is linked to the hygroscopic nature of the molecule. This property attracts water to its polysaccharide structure, forming a three-dimensional network capable of enhancing the viscoelasticity of emulsifying systems [40]. The rheograms revealed that as shear force increased, viscosity tended to decrease, a characteristic of formulations with pseudoplastic flow [14]. This phenomenon occurs because as shear stress rises, the polymer structure aligns along the shear direction, resulting in faster subsequent shear and a decrease in apparent viscosity [19]. Notably, HA exhibits great potential as a carrier molecule for delivering active ingredients, both in topical and transdermal systems. Its viscoelasticity, biocompatibility, biodegradability, and non-allergenic characteristics make it pivotal for the development of emulsions [41].

The pH of a formulation must be compatible with biological tissues to ensure its stability and effectiveness. Additionally, extremely low pH values should be avoided in nanoemulsion systems because they diminish electrostatic repulsion between particles, leading to an increase in droplet size and coalescence [42]. In this study, the pH of the nanoemulsions ranged between 5.80–6.55, which is suitable for topical application [43]. Studies have indicated that pH values close to neutrality (≈6–7) promote an increase in zeta potential in emulsions containing HA. This occurs through the deprotonation of carboxyl (–COO–) and hydroxyl (OH–) groups in the molecular structure of HA, imparting a negative charge to the oil–water interface [14,44,45], consistent with the findings of this study.

High zeta values (>30 mV absolute value) have been proposed as an indicator of physical stability, as they ensure the creation of a high repulsive energy barrier between lipid droplets [46]. Furthermore, in addition to the effect of HA at the droplet interface, the use of a non-ionic surfactant (Cremophor) on the surface also contributed to the adequate absolute zeta value. This is due to the presence of free fatty acids in the surfactant, which facilitate the adsorption of OH– ions from the water onto the surface of the droplets, resulting in a negative charge at pH close to neutrality [14,47]. Based on the smallest droplet size and satisfactory PDI, zeta potential, pH, and viscosity, the nanoemulsions prepared with 10.0% surfactant were found to be promising for the intended purpose and were evaluated for antimicrobial effect.

In the early infection stage, *S. aureus* and *S. pyogenes* are the dominant pathogens involved, while *E. coli* and *P. aeruginosa* are more frequently found when a chronic wound develops [48]. Notably, in addition to antimicrobial resistance, *S. aureus* and *P. aeruginosa* are strains that most express virulence factors affecting skin healing [49]. Although the antimicrobial activity of chamomile is well-documented scientifically, this work found that the incorporation of the extract into the nanoemulsion system (F2) enhanced the antimicrobial effect compared to the isolated extract, against all bacteria tested. Furthermore, in addition to providing an adequate skin distribution system, the emulsification or encapsulation of antimicrobial agents can accelerate their absorption and increase the bioavailability of the active ingredients, thereby enhancing the therapeutic effect [50], justifying the findings of this study.

Additionally, against *S. aureus* and *P. aeruginosa*, the MIC significantly decreased after stabilization of the nanoemulsion with HA (F2 + HA), indicating a possible synergistic effect. According to Zamboni et al. [51], HA can exert a bacteriostatic effect. Therefore, incorporating products with antimicrobial action in systems with HA can potentially generate a synergistic action, inhibiting the multiplication of bacteria and promoting an effective approach to treating topical infections.

Hyaluronic acid plays an integral role in wound healing, facilitating fibrin coagulation, modulating inflammatory response, promoting re-epithelialization, inducing migration and multiplication of dermal fibroblasts, and favoring angiogenesis [52]. Hence, combining the fight against oxidative stress and the antimicrobial effect of *C. recutita* extract, linked to the inherent benefits of the presence of HA in a formulation, leads to the inference that the F2 + HA nanoemulsion has potential for topical application, with a possible auxiliary effect on the healing process. The scope of this study was limited to the development of the nanoemulsion system. In future studies, the physicochemical stability and in vivo wound healing activity should be evaluated.

## 5. Conclusions

*C. recutita* extract, prepared with a 70% hydroalcoholic solution, exhibits in vitro antimicrobial activity against *S. aureus* (MIC = 26.04 mg mL^−1^), *S. pyogenes* (MIC = 1.62 mg mL^−1^), *E. coli* (MIC = 62.50 mg mL^−1^), and *P. aeruginosa* (MIC = 15.62 mg mL^−1^) and antioxidant capacity in DPPH, ABTS, and FRAP assays (22.32 μM_Trolox_, 778.59 µmol_Trolox_ g_ext_^−1^ and 1085.70 µmol_Fe_^2+^ g_ext_^−1^, respectively). This antimicrobial activity is further enhanced by incorporating the extract into a nanoemulsion system stabilized with HA. Based on physicochemical data, F2 + HA, formulated with 10.0% surfactant agent, 1.0% HA, and enriched with *C. recutita* extract, demonstrates homogeneous droplet size and PDI, a pH compatible with the skin, and satisfactory zeta potential and viscosity. These attributes make it suitable for topical application, potentially assisting in healing processes.

## Figures and Tables

**Table 1:** Composition of nanoemulsion systems (%, *w*/*w*).

Nanoemulsion System	Pre-Emulsion without *C. recutita*	Pre-Emulsion with *C. recutita*	Cremophor	Saline Solution (0.1 M)	HA
C1	20.0	-	7.5	72.5	-
C1 + HA	20.0	-	7.5	71.5	1.0
C2	20.0	-	10.0	70.0	-
C2 + HA	20.0	-	10.0	69.0	1.0
C3	20.0	-	12.5	67.5	-
C3 + HA	20.0	-	12.5	66.5	1.0
F1	-	20.0	7.5	72.5	-
F1 + HA	-	20.0	7.5	71.5	1.0
F2	-	20.0	10.0	70.0	-
F2 + HA	-	20.0	10.0	69.0	1.0
F3	-	20.0	12.5	67.5	-
F3 + HA	-	20.0	12.5	66.5	1.0

C: nanoemulsion system without *C. recutita* extract. F: nanoemulsion system loaded with *C. recutita* extract. HA: hyaluronic acid for stabilization.

**Table 2:** Total flavonoid content (µg_QUE_ mg_ext_^−1^) in *C. recutita* extracts as a function of the extractive alcohol content.

Method	Alcohol Content (%)	Total Flavonoid Content (µg_QUE_ mg_ext_^−1^)
Vortex Extraction	50.0	165.08 ± 2.55 ^c^
	60.0	195.53 ± 4.03 ^c^
	70.0	280.41 ± 2.19 ^a^
	80.0	275.10 ± 3.38 ^ab^
	90.0	233.33 ± 1.28 ^b^
	100.0	176.73 ± 0.57 ^c^
Infusion	0.0	117.08 ± 0.89 ^d^

Mean value (*n* = 3) ± standard deviation. Averages showing the same letter within columns do not differ significantly from each other (*p* < 0.05) according to the ANOVA with Tukey’s test.

**Table 3:** Phytochemical identification of *C. recutita* extract by UHPLC-MS.

Compound	*m/z*	Retention Time (min)	% *
Spireoside	463.08	14.50	29.47
Apiin	563.09	17.24	12.62
Corymbosin	357.09	11.03	6.34
Isoxanthohumol	353.09	17.12	6.21
Kaempferol 3-O-neohesperidoside	593.12	18.53	6.05
Isosakuranin	447.13	3.36	4.10
Xanthohumol	353.13	20.10	3.58
Aspalathin	451.13	9.14	3.14
Scaposin	389.08	8.05	3.12
Salicylic acid	137.02	8.10	2.95
Neohesperidin	609.17	3.39	2.93
Pseudobaptigenin	281.04	3.93	2.84
Humulone	361.20	11.04	2.67
Morin	299.02	19.15	2.55
Apigenin	269.10	11.88	2.55
Kaempferol-3-O-glucoside	447.08	16.09	1.62
4′,7-dimethoxy-5-hydroxyflavanone	299.09	3.23	1.38
5,7-dihydroxy-2′-methoxyflavone	283.06	3.34	1.14
Kaempferide	299.05	3.21	0.90
Chrysin	253.05	3.39	0.90
4-Malonyl ononin	267.06	3.11	0.83
3-Ara-28-Glu hederagenin	811.44	3.39	0.82
Luteolin 4′-O-glucoside	447.09	15.32	0.38
Sinapoyl malate-4′-methyl ester	353.06	4.22	0.37
6-Methoxyluteolin	315.05	10.52	0.29
Naringin	579.17	12.56	0.20
Sinapoyl malate-1′-methyl ester	353.07	3.16	0.07
Free flavonoids			65.62
Glycosylated flavonoids			15.63
Cinnamic acid derivatives			6.25
Organic acids			6.25
Cyclic ketone			3.13
Terpenes			3.12

* Percentages of phytochemical compounds were calculated based on the total amount of identified compounds.

**Table 4:** Influence of different surfactant concentrations on the size, PDI, pH, viscosity, and zeta potential of nanoemulsion systems loaded with *C. recutita* and stabilized with HA.

Formulation	Droplet Size (nm)	PDI	pH	Viscosity (mPa s) *	Zeta Potential (mV) **
C1	254.09 ± 14.13 ^a^	0.296 ± 0.015 ^ab^	6.37 ± 0.15 ^bc^	339.80 ± 20.35 ^g^	-
C1 + HA	258.95 ± 3.18 ^a^	0.262 ± 0.007 ^bcd^	6.28 ± 0.03 ^cde^	2316.97 ± 45.54 ^e^	-
C2	139.12 ± 10.49 ^c^	0.118 ± 0.006 ^g^	6.55 ± 0.14 ^ab^	268.83 ± 5.46 ^g^	−41.93 ± 1.67 ^c^
C2 + HA	142.19 ± 16.54 ^c^	0.196 ± 0.006 ^f^	6.28 ± 0.02 ^cde^	2543.87 ± 77.01 ^d^	−51.83 ± 1.27 ^b^
C3	231.45 ± 9.99 ^ab^	0.244 ± 0.010 ^cde^	6.55 ± 0.05 ^ab^	338.73 ± 15.80 ^g^	-
C3 + HA	248.32 ± 8.80 ^a^	0.279 ± 0.009 ^abc^	6.33 ± 0.01 ^bcd^	2969.07 ± 148.38 ^b^	-
F1	254.09 ± 4.20 ^a^	0.278 ± 0.008 ^abc^	5.80 ± 0.08 ^g^	306.93 ± 17.38 ^g^	-
F1 + HA	262.63 ± 11.50 ^a^	0.218 ± 0.011 ^ef^	6.09 ± 0.00 ^ef^	2039.97 ± 40.26 ^f^	-
F2	139.70 ± 3.96 ^c^	0.116 ± 0.007 ^g^	6.50 ± 0.05 ^abc^	295.93 ± 37.89 ^g^	−51.6 ± 0.3 ^b^
F2 + HA	173.85 ± 1.91 ^bc^	0.144 ± 0.006 ^g^	6.52 ± 0.02 ^ab^	2840.23 ± 180.79 ^bc^	−55.2 ± 0.4 ^a^
F3	231.45 ± 12.09 ^ab^	0.235 ± 0.007 ^de^	5.97 ± 0.16 ^fg^	330.03 ± 32.58 ^g^	-
F3 + HA	245.57 ± 8.73 ^ab^	0.255 ± 0.008 ^cde^	6.12 ± 0.04 ^def^	3515.27 ± 197.25 ^a^	-

Results are expressed as mean ± standard deviation (*n* = 3). Different letters within the same column represent significant differences (*p* < 0.05). C: nanoemulsion system without *C. recutita* extract. F: nanoemulsion system loaded with *C. recutita* extract. HA: hyaluronic acid for stabilization. * Viscosity at 5 rpm. ** Only the most promising formulations were evaluated for zeta potential.

**Table 5:** Minimum inhibitory concentration (mg mL^−1^) of the pure *C. recutita* extract incorporated into a nanoemulsion system stabilized with HA.

	*S. aureus*	*S. pyogenes*	*E. coli*	*P. aeruginosa*
*C. recutita* extract	26.04 ± 9.02	1.62 ± 0.56	62.50 ± 0.00	15.62 ± 0.00
C2	-	-	-	-
C2 + HA	-	-	-	-
F2 *	250.00 ± 0.00	250.00 ± 0.00	250.00 ± 0.00	250.00 ± 0.00
F2 + HA *	166.66 ± 72.16	250.00 ± 0.00	250.00 ± 0.00	166.66 ± 72.16
Neomycin	4.88 × 10^−3^ ± 0.00	4.88 × 10^−3^ ± 0.00	39.06 × 10^−3^ ± 0.00	9.76 × 10^−3^ ± 0.00

* Values correspond to the amount (in mg mL^−1^) of nanoemulsion required for antimicrobial effect. (-) indicates no activity. C: nanoemulsion system without *C. recutita* extract. F: nanoemulsion system loaded with *C. recutita* extract. HA: hyaluronic acid for stabilization.

## Data Availability

Data are contained within the article.
